# Diagnostic performance of PAX1 methylation as a biomarker for cervical lesions: a clinical study and meta-analysis

**DOI:** 10.1080/07853890.2025.2583319

**Published:** 2025-11-12

**Authors:** Man Yin, Jiahui Dai, Ronghe Sun, Yunfei Wang

**Affiliations:** a Department of Gynecologic Oncology, Zhongnan Hospital of Wuhan University, Wuhan, Hubei Province, China; b Department of Gynecologic, Affiliated Hospital of Jining Medical University, Jining, Shandong, China; c Center of Obstetrics and Gynecology, Peking University Shenzhen Hospital, Shenzhen, P. R. China; d Institute of Obstetrics and Gynecology, Shenzhen PKU-HKUST Medical Center, Shenzhen, P. R. China; e Shenzhen Key Laboratory on Technology for Early Diagnosis of Major Gynecologic Diseases, Shenzhen, P. R. China

**Keywords:** Cervical cancer, cervical lesion, paired boxed gene 1, DNA methylation, diagnostic performance

## Abstract

**Objective:**

To evaluate the diagnostic performance of PAX1 gene methylation in the detection of cervical lesions and assess its potential clinical application in cervical cancer screening through both a single-centre study and a meta-analysis.

**Methods:**

A retrospective analysis was conducted on 329 patients who underwent concurrent ThinPrep cytologic test (TCT), high-risk HPV testing and PAX1 methylation analysis at the Affiliated Hospital of Jining Medical University. The diagnostic accuracy of PAX1 methylation for detecting high-grade squamous intraepithelial lesions (HSIL) and cervical squamous cell carcinoma (CSCC) was assessed. Additionally, we performed a systematic review and meta-analysis, which included seven eligible studies from Asian populations. Pooled diagnostic metrics were calculated using a bivariate mixed-effects model. Heterogeneity and publication bias were evaluated using Cochran’s Q test, I^2^ statistics and Deeks’ funnel plot asymmetry test.

**Results:**

PAX1 methylation values declined with increasing severity of cervical lesions. In clinical samples, the sensitivity and specificity for detecting CSCC were 81.3% and 94.2%, respectively. Meta-analysis yielded a pooled sensitivity of 0.87 (95% CI: 0.79–0.92), specificity of 0.75 (95% CI: 0.52–0.89) and an area under the curve (AUC) of 0.89. The diagnostic odds ratio was 20, indicating strong discriminatory performance.

**Conclusion:**

PAX1 methylation demonstrates high diagnostic accuracy for cervical cancer and is a promising noninvasive biomarker for screening and triage. Further studies are needed to validate its clinical utility in diverse populations and to explore its potential role in monitoring lesion progression or regression, particularly in women managed with conservative approaches.

## Key messages

1. We analysed the clinical data to verify that paired boxed gene 1 (PAX1) methylation is a potential biomarker of cervical lesions.
2. We analysed the literature related to PAX1 methylation and summarized its diagnostic performance.
3. The clinical and literature analysis confirmed the advantages and possible differences of PAX1 methylation diagnosis.

## 1. Introduction

Cervical cancer is the fourth most common cancer in women worldwide [1], with an estimated 660,000 new cases and 350,000 deaths in 2022. The highest burden is observed in low- and middle-income countries, where access to effective screening and treatment is often limited. Persistent infection with high-risk human papillomavirus (HPV), which is a necessary cause of cervical cancer [2–4], typically develops through a slow progression from normal epithelium to low-grade squamous intraepithelial lesions (LSIL) and high-grade squamous intraepithelial lesions (HSIL), and eventually to invasive cancer [5]. This long precancerous phase provides an opportunity for early detection and intervention.

Current cervical cancer screening strategies are increasingly shifting from cytology-based methods to primary HPV testing, given its higher sensitivity for detecting women at risk of cervical precancer and cancer. In this approach, cytology methods such as the ThinPrep cytologic test (TCT) are commonly used as a triage tool for HPV-positive women. Although HPV testing offers excellent sensitivity, its specificity is limited due to the high prevalence of transient infections that typically resolve without clinical consequence. As approximately 95% of cervical cancer cases are attributable to persistent infection with oncogenic HPV types [6], there is a growing need for effective triage strategies and novel biomarkers that can better distinguish women at true risk of progression. This would improve the precision of screening programs and reduce unnecessary follow-up procedures.

DNA methylation is a key epigenetic mechanism involving the enzymatic addition of methyl groups to cytosine residues in CpG dinucleotides, typically within gene promoter regions, without altering the underlying DNA sequence. This process plays a critical role in regulating gene expression and is closely linked to the development and progression of many cancers [7]. The paired box gene 1 (PAX1), located on chromosome 20p11, encodes a transcription factor with a paired domain (PD) and an octapeptide domain (OP) [8]. It functions as a tumour suppressor and is frequently silenced through promoter hypermethylation in various malignancies. Lai et al. [9] first demonstrated that PAX1 is significantly more methylated in cervical cancer tissue compared to normal cervical epithelium.

In addition to PAX1, other methylation-based biomarkers such as SOX1 and ZNF582 have shown moderate-to-high sensitivity and specificity for detecting HSIL and cervical cancer. However, their ability to distinguish low-grade lesions remains limited, and combining them with PAX1 may improve overall diagnostic coverage [9–11]. In recent years, multiple studies have reported promising results for PAX1 methylation as a biomarker in cervical cancer screening [12,13].

The aim of this study was to evaluate the diagnostic performance of PAX1 methylation in detecting cervical lesions, including HSIL and cervical squamous cell carcinoma (CSCC), by using real-time PCR in a clinical cohort. To address the potential limitations of single-centre data, including geographic or methodological variability, we also conducted a meta-analysis of published studies to assess the broader applicability and diagnostic value of PAX1 methylation across different populations and testing platforms.

## 2. Materials and methods

### 2.1. Clinical studies

#### 2.1.1. General information

We conducted a retrospective cohort study involving patients who underwent cervical cancer screening at the Department of Gynecology, Affiliated Hospital of Jining Medical University, between November 2019 and October 2021. A total of 329 patients were included: 145 with normal histology, 101 with low-grade squamous intraepithelial lesions (LSIL), 67 with high-grade squamous intraepithelial lesions (HSIL) and 16 with cervical squamous cell carcinoma (CSCC). The study was reviewed and approved in advance by the ethics committee of the Affiliated Hospital of Jining Medical University (ethic approval No. 2022C025).

Inclusion criteria were: (1) availability of histological diagnosis based on cervical biopsy and corresponding PAX1 methylation test results; (2) non-pregnant status; (3) absence of immunodeficiency; (4) no prior HPV vaccination and (5) no history of cervical surgery or chemo-/radiotherapy for cervical cancer.

Exclusion criteria were: (1) concurrent gynaecologic malignancies (e.g. endometrial or ovarian cancer); (2) cervical treatment (e.g. cryotherapy, laser therapy) within 3 months before sampling; (3) missing methylation test results and (4) incomplete clinical data or unclassifiable histology.

Patients were stratified into four groups based on the histopathological diagnosis of cervical biopsy specimens: normal cervix (NC), LSIL, HSIL and CSCC. All specimens were independently reviewed by two experienced pathologists; discrepancies were resolved through consensus with a third reviewer.

All participants provided written informed consent. The study was conducted in accordance with the Declaration of Helsinki and relevant institutional guidelines.

#### 2.1.2. ThinPrep cytologic test (TCT)

A specialized cervical cytology brush was inserted approximately 1 cm into the cervical canal and rotated five times clockwise. The brush head was then placed into a liquid-based cytology preservation solution. Cytology slides were prepared using a ThinPrep automated processor, stained with the Papanicolaou (Pap) stain and examined under a light microscope.

#### 2.1.3. HPV testing

HPV DNA testing was performed in parallel with TCT using the Hybrid Capture 2 (HC2) method. Exfoliated cervical cells were collected from the transformation zone using a cytobrush and suspended in a dedicated fixative solution. HPV DNA was detected through nucleic acid hybridization; results were considered positive when HPV-DNA levels exceeded 1.0 pg/mL.

#### 2.1.4. PAX1 methylation analysis

Genomic DNA was extracted from exfoliated cervical cells using the QIAamp DNA Mini Kit. DNA purity was assessed with a NanoDrop 2000 spectrophotometer. Bisulphite conversion was performed using a commercial kit to convert unmethylated cytosine to uracil. Methylation-specific primers targeting the CpG island in the PAX1 promoter were used in a real-time PCR to quantify PAX1 methylation levels. ΔCp values (Cp_FAM-Cp_VIC) and fluorescence ratios (FAM/VIC) were calculated for each sample.

#### 2.1.5. Histopathological examination

Following cervical cancer screening, colposcopic-directed cervical biopsies were obtained. Tissue specimens were fixed in 10% neutral-buffered formalin for 24 h, paraffin-embedded, sectioned, and stained with haematoxylin and eosin (H&E). Additional immunohistochemical staining was performed as needed. Slides were examined by experienced pathologists and classified into four diagnostic categories: normal/inflammatory (negative), LSIL, HSIL and CSCC.

#### 2.1.6. Statistical analysis

Statistical analyses were conducted using SPSS version 21.0 (Chicago, IL, USA). Continuous variables were analysed to assess the diagnostic accuracy of PAX1 methylation, including sensitivity and specificity calculations. A *p*-value < 0.05 was considered statistically significant.

### 2.2. Meta-analysis

#### 2.2.1. Retrieval strategy

To further evaluate the diagnostic performance of PAX1 methylation in cervical lesions, we also searched the database to perform a meta-analysis, which was conducted according to the statement of the Preferred Reporting Items for Systematic reviews and Meta-Analyses (PRISMA) published in 2020. We searched three databases, PubMed, Cochrane and Embase, before September 1, 2023. The following search formula was used: [‘methylation’ or ‘DNA methylation’ or ‘methylation marker’] and [‘Uterine Cervical Neoplasms’ or ‘Cervical Neoplasms’ or ‘Cervical Cancer’ or ‘cancer of the cervix’ or ‘uterine cervical dysplasia’ or ‘Squamous Intraepithelial Lesion’] and [‘Paired box PAX1’ or ‘PAX1’].

#### 2.2.2. Acceptance and discharge standard

We analysed all relevant references and included the literature according to the following criteria:

1. The diagnostic performance of PAX1 methylation in the diagnosis of high-grade squamous intraepithelial neoplasia (HSIL) or cervical neoplasia was evaluated; and
2. The study clearly mentions diagnostic information such as sample size, sensitivity and specificity.

The literature was excluded based on the following criteria:

1. Nonclinical research, such as basic research, animal studies, review articles, etc.;
2. Unclear sample size;
3. The control group was unclear;
4. The methylation of the subject diagnosed with other types of tumours;
5. The gold standard was not tissue biopsy pathology; and
6. Missing diagnostic information such as sensitivity and specificity.

#### 2.2.3. Data extraction and quality assessment

All included studies were extracted independently by two researchers for the following information: year of publication, first author, sample size, study design, extent of cervical lesions, test method, diagnostic results and PAX1 methylation levels. The second version of the Quality Assessment of Diagnostic Accuracy Studies (QUADAS-2) [14] was used to assess the quality of each study included. Each domain was assessed according to risk of bias and applicability concerns, and any differences of opinion were resolved by consensus of the panel.

#### 2.2.4. Data analysis

Statistics were conducted with Revman 5.4 (Review Manager), Stata 16.0 (Stata Corporation, College Station, TX, USA) and MetaDisc 1.4 (XI Cochrane Colloquium, Barcelona, Spain) software. The included studies were evaluated using the QUADAS-2 assessment tool, and bivariate mixed-effects models were used to summarize sensitivity, specificity, positive likelihood ratio (PLR), negative likelihood ratio (NLR) and diagnostic odds ratio (DOR). Summary ROC (SROC) curves and their corresponding 95% confidence intervals were generated. The combined diagnostic indices were calculated using a random effects model. Heterogeneity among articles was assessed by I^2^ statistics and Cochran’s Q test, with P heterogeneity <0.10 and I^2^ > 50% indicating heterogeneity [15]. In addition, influence assays and meta-regression were used to track potential sources of study heterogeneity. To assess the potential publication bias, the Deeks funnel plot asymmetry test was used.

## 3. Results

### 3.1. Clinical case data analysis

**Figure 1.**
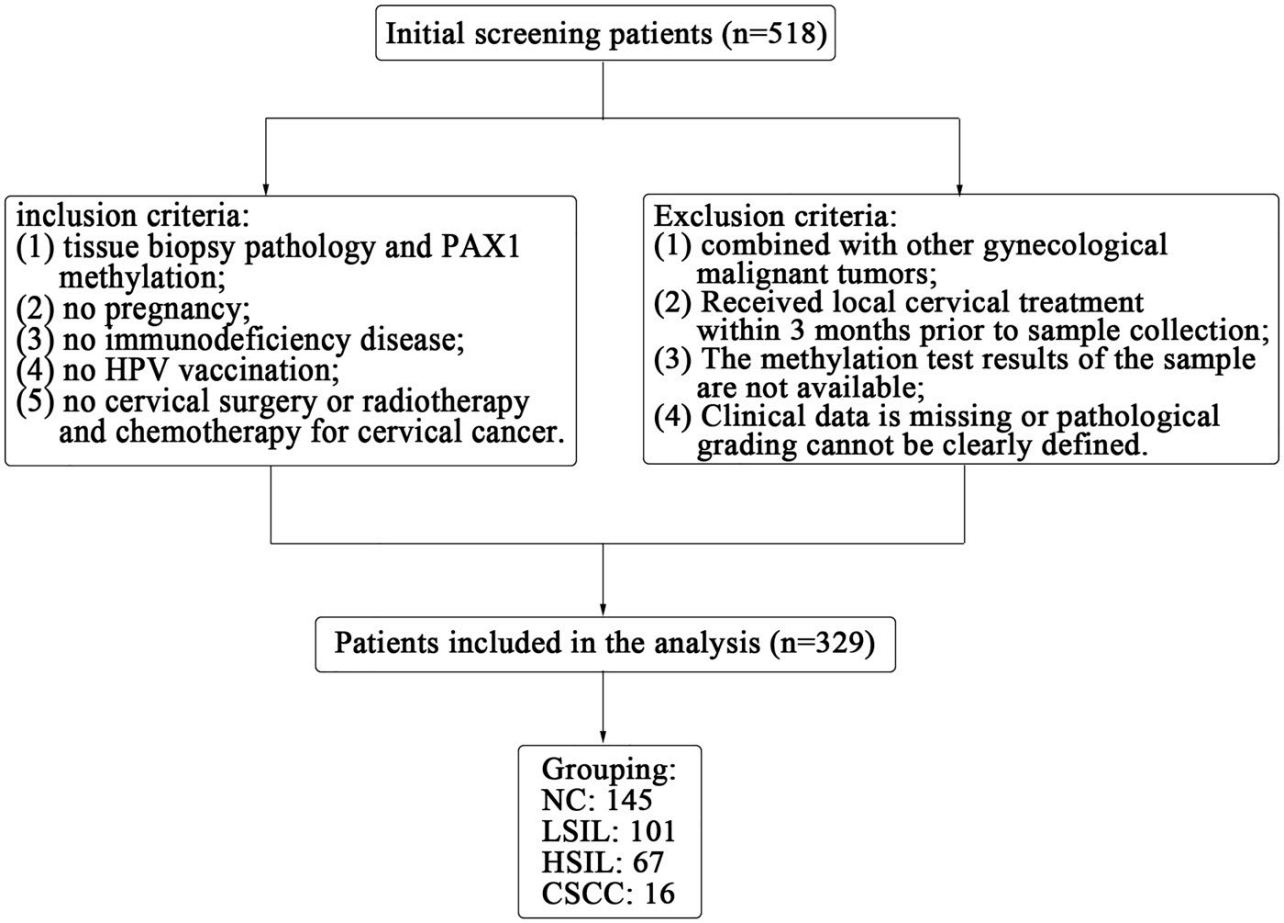
Patient inclusion flowchart.

**Figure 2.**
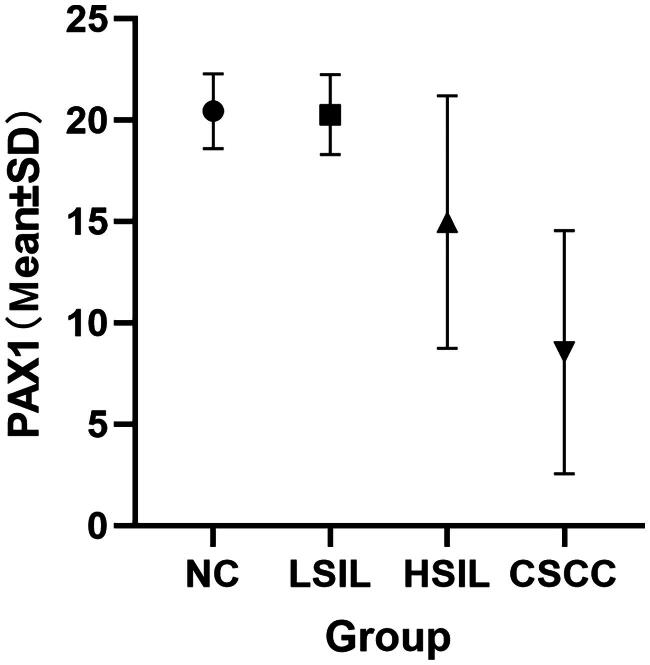
Quantitative comparison of PAX1 methylation in different groups of exfoliated cervical cells.

A total of 329 patients were included in the study (Figure 1), classified histologically as follows: 145 with normal cervix (NC), 101 with low-grade squamous intraepithelial lesions (LSIL), 67 with high-grade squamous intraepithelial lesions (HSIL) and 16 with cervical squamous cell carcinoma (CSCC). Patients with more severe cervical lesions tended to be older, with mean ages of 37.2 years in the LSIL group, 39.5 years in the HSIL group and 46.2 years in the CSCC group (*p* < 0.01). Conversely, the PAX1 methylation Cp values significantly decreased with increasing lesion severity: mean Cp values were 20.58 ± 1.36 in NC, 20.37 ± 1.69 in LSIL, 14.96 ± 6.23 in HSIL and 8.56 ± 6.00 in CSCC (*p* < 0.0001). There was no statistically significant difference in methylation values between the NC and LSIL groups (Table 1, Figure 2). HPV positivity rates exceeded 90% across all groups, including those with normal histology (93.8%) and LSIL (95.0%). This high HPV positivity rate in women without high-grade disease may lead to unnecessary colposcopic referrals. In contrast, PAX1 methylation analysis provided better stratification. Based on the data, a Cp value threshold of 18.68 was proposed: patients with values below this cut-off were more likely to have HSIL or worse and should be considered for colposcopy. The proportion of HPV16/18-positive cases increased with lesion severity, representing 59.1% of HSIL and 81.3% of CSCC cases (*p* < 0.05). Additionally, the ThinPrep cytologic test (TCT) demonstrated limited sensitivity for detecting HSIL and CSCC, with HSIL cytology results found in only 17.0% and 21.4% of histologically confirmed HSIL and CSCC cases, respectively (*p* < 0.0001) (Table 1). These findings underscore the limitations of cytology and support the potential value of PAX1 methylation as a triage biomarker.

**Table 1.** Characteristics and gene methylation of participants.

Variable	NC (*n* = 145)	LSIL (*n* = 101)	HSIL (*n* = 67)	CSCC (*n* = 16)	*p* value
Age	41.9 ± 11.3	37.2 ± 11.3	39.5 ± 10.6	46.2 ± 10.5	*p* < 0.01
C_P_ value	20.6 ± 1.4	20.4 ± 1.7	15.0 ± 6.2	8.6 ± 6.0	*p* < 0.0001
HPV positive rate	93.8%	95.0%	98.5%	87.5%	0.21
Positive rate of 16、18 subtypes	44.4%	39.8%	59.1%	81.3%	*p* < 0.05
Proportion of HPV positive subtypes 16 and 18	47.4%	41.9%	60.0%	92.9%	*p* < 0.01
TCT, %					
NC, ASC-US, ASC-H	87.8%	74.4%	69.5%	71.4%	*p* < 0.05
LSIL	10.7%	24.4%	13.6%	7.1%	*p* < 0.05
HSIL	1.5%	1.1%	17.0%	21.4%	*p* < 0.0001

**Figure 3.**
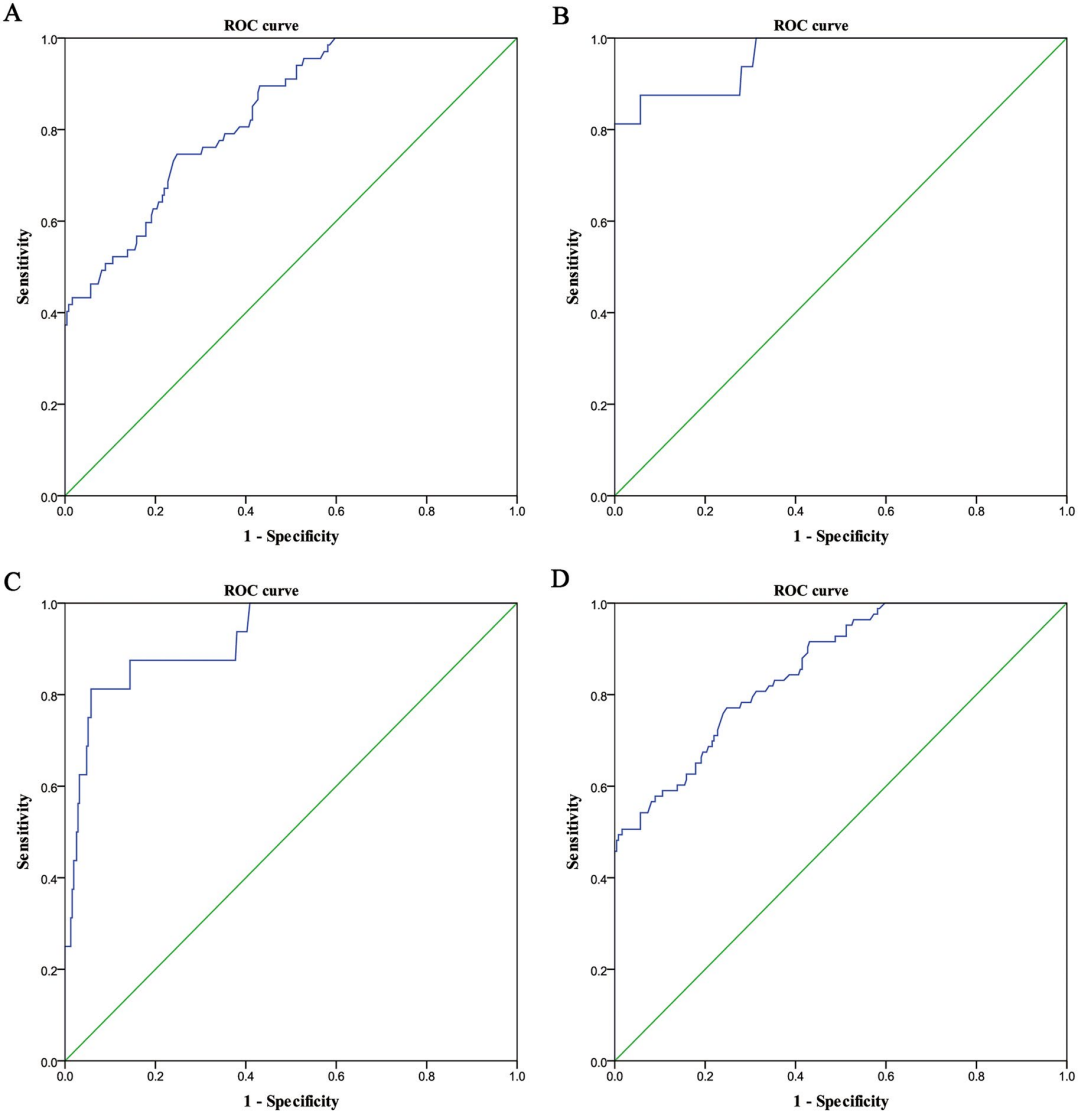
ROC curves for PAX1 quantitative methylation diagnostic thresholds. A: HSIL vs LSIL+NC; B: CSCC vs LSIL+NC; C: CSCC vs HSIL+LSIL+NC; D: HSIL+CSCC vs LSIL+NC.

Since there was no significant difference in PAX1 methylation values between patients with normal cervical biopsy results and those with LSIL, we performed receiver operating characteristic curve (ROC) analysis of methylation values for patients with HSIL and CSCC. The results are shown in Figure 3 and Table 2. The cut-off values of PAX1 methylation detection for patients with HSIL and CSCC were 20.30 and 9.34, respectively, reflecting very high sensitivities for both. The diagnostic specificities for CSCC patients was stronger, and the positive likelihood ratio and diagnostic odds ratio were higher than those of HSIL, significantly improving the detection of the disease and providing stronger cancer detection ability for patients with CSCC.

**Table 2.** Diagnostic indices of PAX1 for the diagnosis of HSIL and CSCC.

	AUC	95%CI	Cut-off value	Sensitivity	Specificity	PLR	NLR	DOR
HSIL vs. LSIL+NC	0.83	0.78-0.89	20.30	0.75	0.75	3.01	0.34	8.90
CSCC vs. LSIL+NC	0.96	0.91-1.00	19.01	0.88	0.94	15.35	0.13	115.42
CSCC vs. HSIL+LSIL+NC	0.92	0.86-0.99	9.34	0.81	0.94	14.02	0.20	70.44
HSIL+CSCC vs LSIL+NC	0.86	0.81-0.90	20.30	0.77	0.75	3.11	0.31	10.19

Abbreviations: AUC: Area Under Curve; PLR: Positive Likelihood Ratio; NLR: Negative Likelihood Ratio; DOR: Diagnostic Odds Ratio.

### 3.2. Meta-analysis

#### 3.2.1. Study characteristics and quality

**Figure 4.**
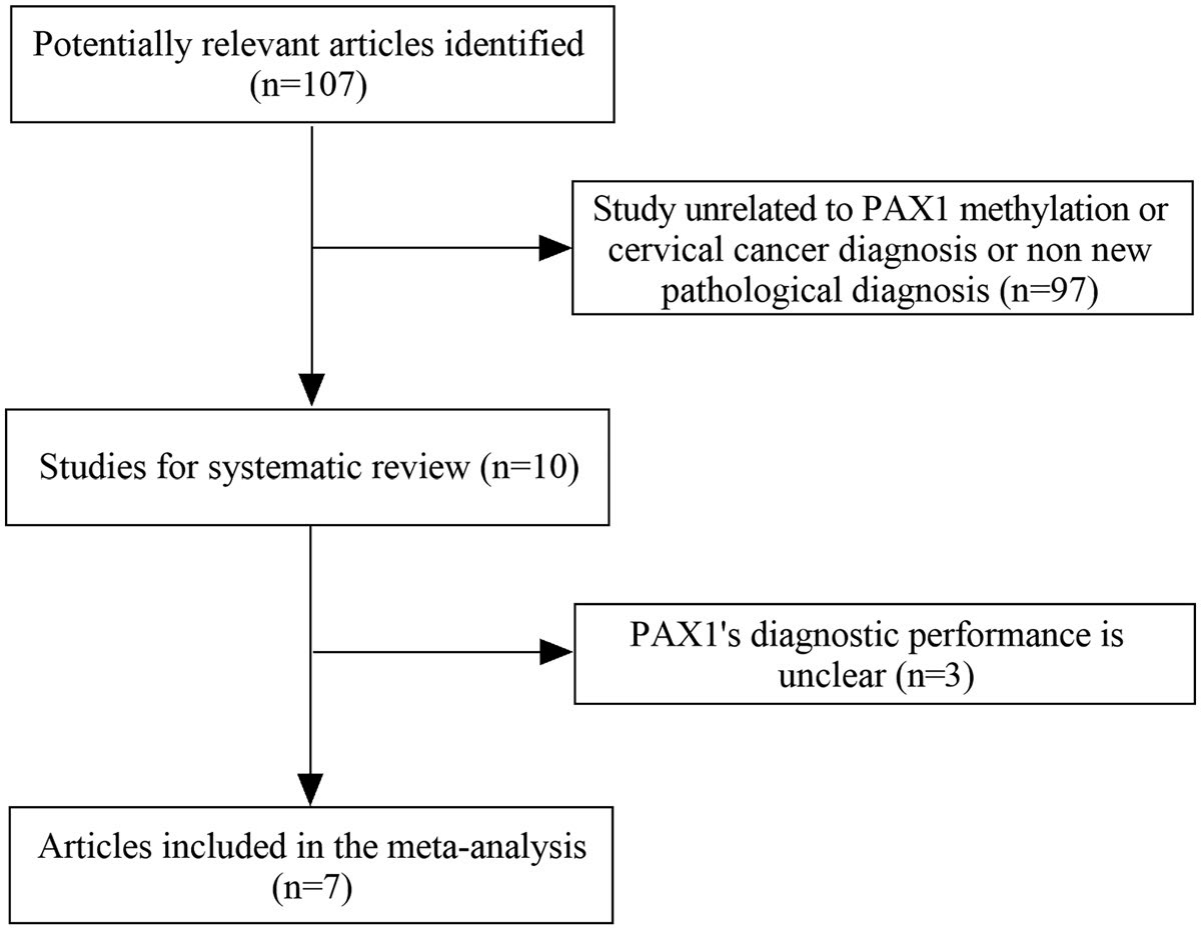
PRISMA Flow chart of the data search.

**Figure 5.**
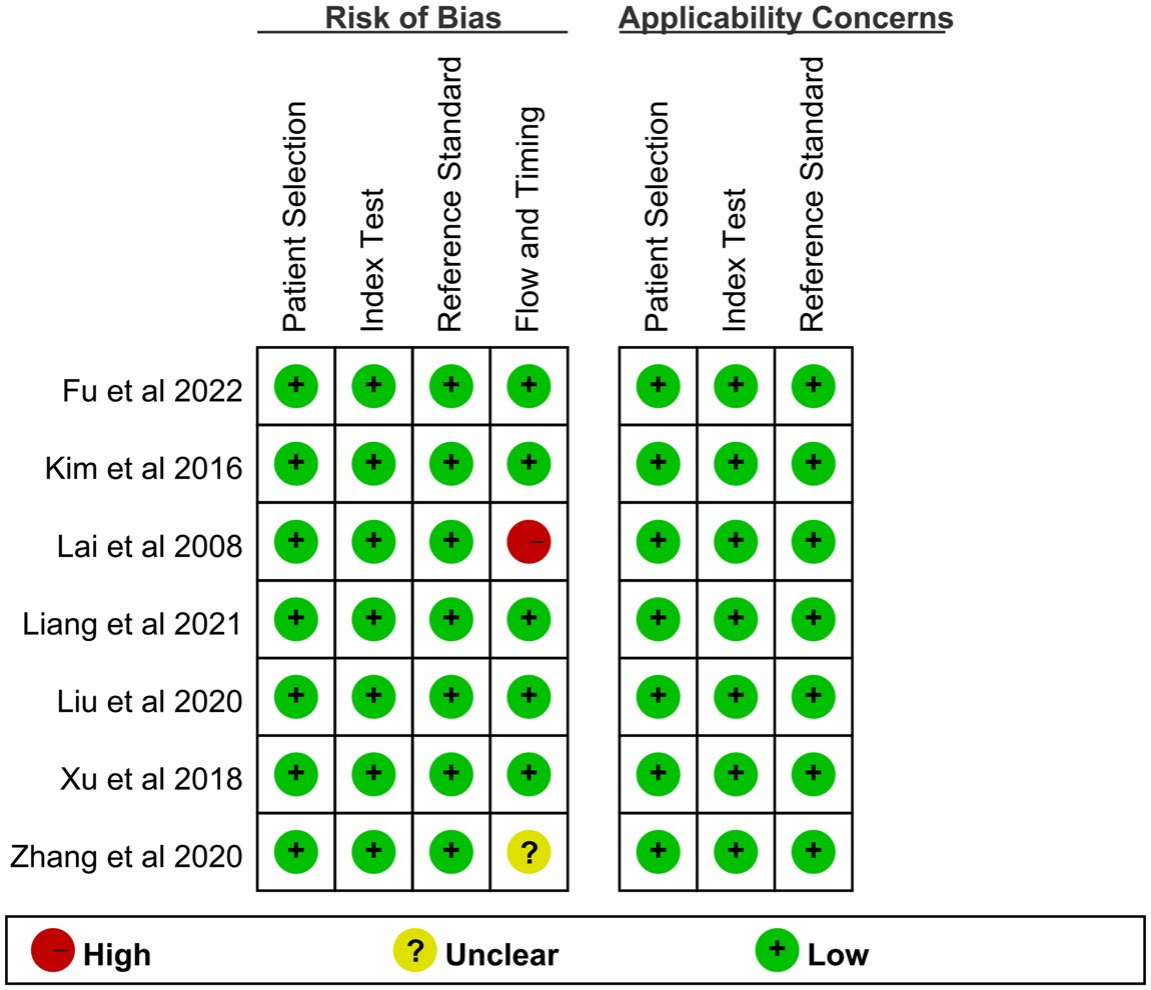
Summary of assessment of the included studies analyzed using the QUADAS tool: proportion of studies with low (yes), mediate (unclear), and high risk of bias (no). QUADAS, quality assessment for studies of diagnostic accuracy.

According to the predetermined criteria, 107 studies were retrieved from the electronic database. As shown in Figure 4, 97 of them were excluded because they were not related to the clinical study of PAX1 methylation or the diagnosis of cervical lesions. After the evaluation of the full text, three studies were excluded because the diagnostic performance of methylation was unclear and the pathological diagnostic criteria were not up to date [16–18]. Finally, seven studies were included in the meta-analysis. Table 3 describes the main characteristics of each included study. All seven studies were performed in Asia, six were performed in China, and one was performed in Korea; the diagnostic gold standard for all studies was histopathology. We assessed the quality of each included study using the QUADAS-2 assessment tool [14], and as shown in Figure 5, all seven studies showed a low risk of bias, indicating a relatively high overall quality of the included analyses.

**Table 3.** Main characteristics of the included PAX1 methylation studies for the diagnosis of cervical lesions.

Author	Year	Study location	Patient size	Number of CSCC patients	Method	Cut-off value
Liu et al. [28]	2020	China	54	14	BSP	based on ROC
Liang et al. [29]	2021	China	105	31	BSP	based on ROC
Zhang et al. [13]	2020	China	487	30	QMSP	based on ROC
Lai et al. [9]	2008	China Taiwan	170	22	MSP/BSP	–
Fu et al. [10]	2022	China	277	39	QMSP	based on ROC
Xu et al. [30]	2018	China	121	27	BSP	based on ROC
Kim et al. [31]	2016	Korea	205	48	BSP	based on ROC

Abbreviations: BSP: Bisulfite Sequencing PCR; MSP: Methylation-specific PCR; QMSP: Quantitative Methylation-Specific PCR.

#### 3.2.2. Heterogeneity

As shown in Table 4, heterogeneity related to threshold and non-threshold effects was assessed using MetaDisc 1.4 software. The *p*-value for Spearman’s correlation coefficient was 0.645 (> 0.05), indicating no significant heterogeneity due to threshold effects in the PAX1 methylation assay. However, Cochran’s Q test revealed a Q value of 23.17 (*p* < 0.01) and an I^2^ value greater than 50%, suggesting the presence of heterogeneity due to non-threshold effects. This heterogeneity may be attributed to variations in detection methods and differences in study population. Among the included studies, five employed bisulphite sequencing PCR (BSP), while two used quantitative methylation-specific PCR (QMSP), the latter generally offering higher sensitivity. In addition, some studies included participants with a broader age range, which could contribute to the inter-study variability.

**Table 4.** Heterogeneity assessment of the individual pooled studies.

Analysis	The value of Spearman correlation coefficient	The value of Cochran’s-Q test	I^2^(%)	Heterogeneity	
				Threshold effect	Non-threshold effect
PAX1	0.214	23.17	74.1	No	Yes
	*p* = 0.645	*p* = 0.0007			

#### 3.2.3. Diagnostic performance

**Figure 6.**
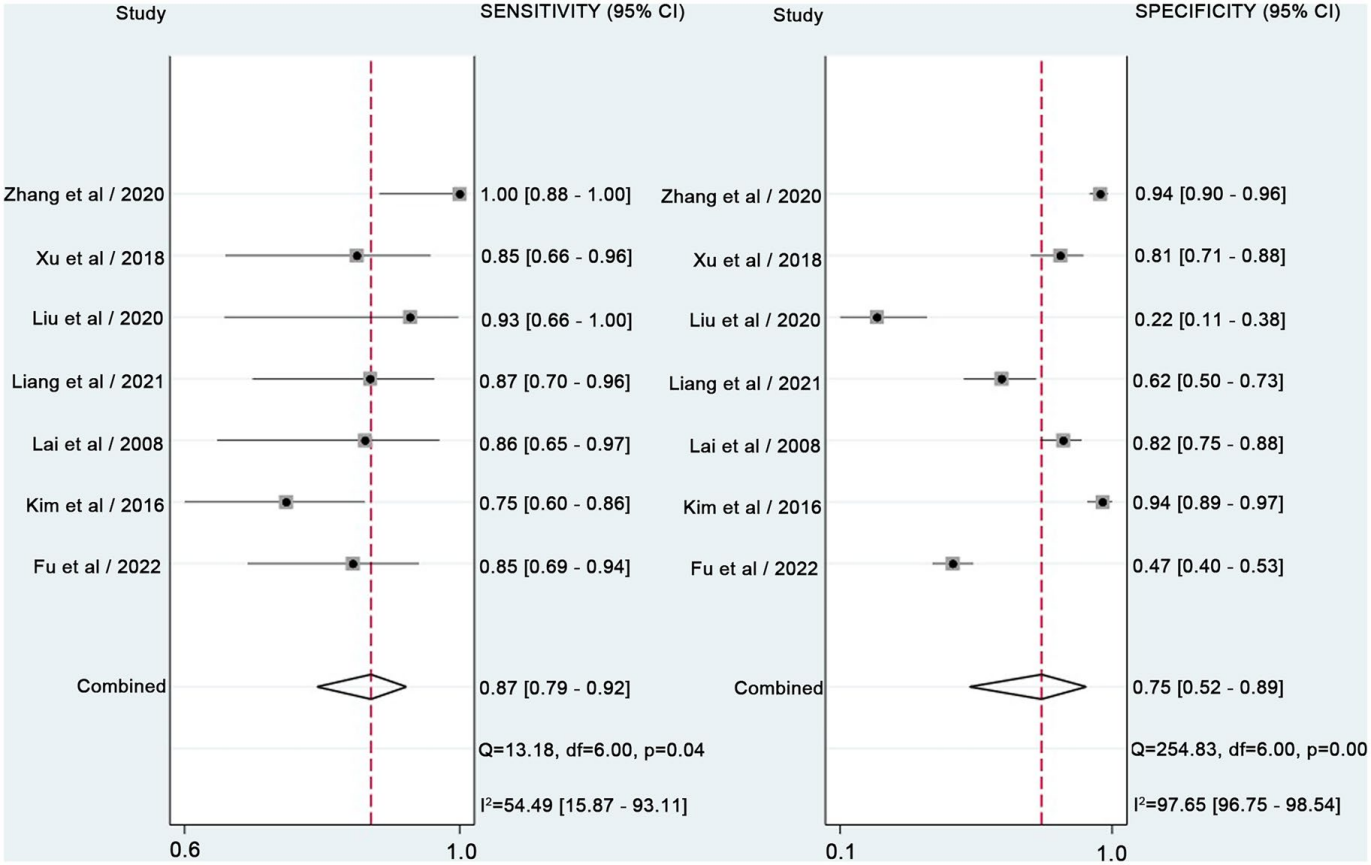
Forest plots of the pooled sensitivity and specificity for PAX1 methylation. Only first author of each study was given. Sensitivity and specificity were given with CI. CIs = confidence intervals.

**Figure 7.**
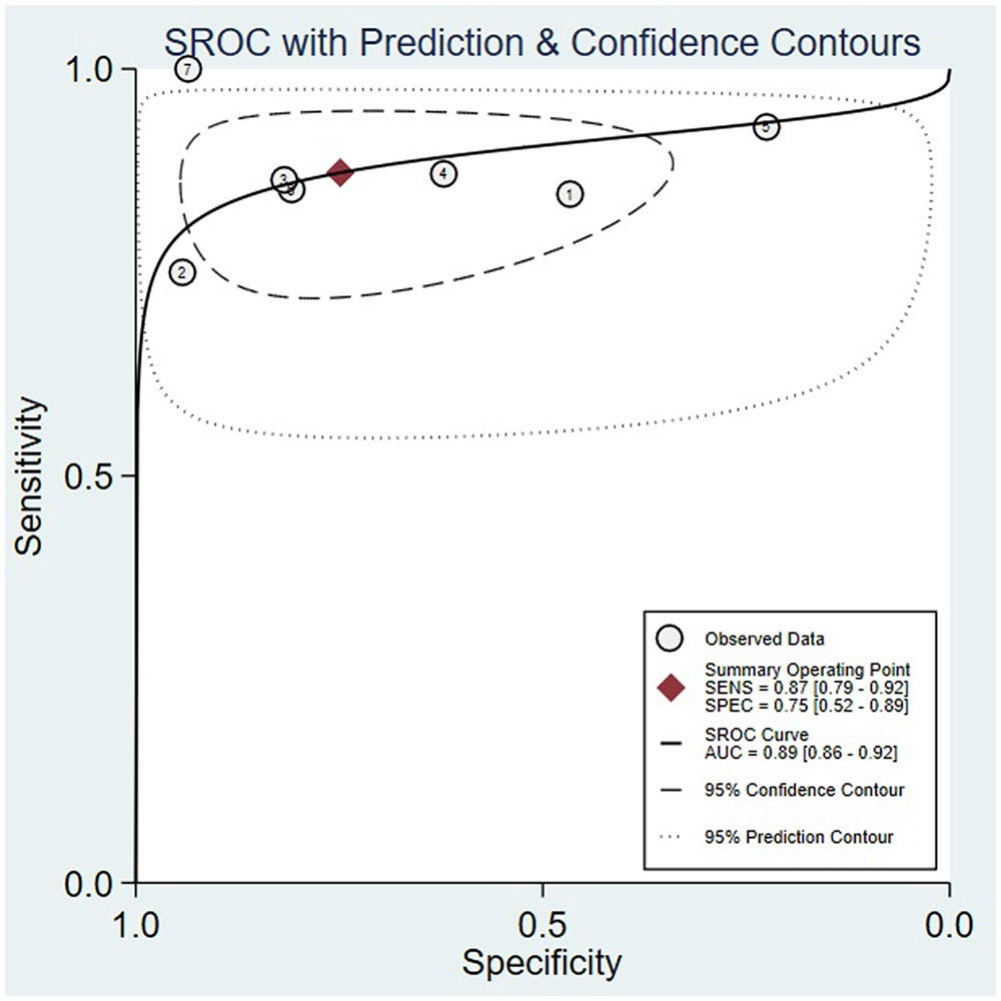
The SROC curves of the pooled individual analyses.

Due to significant heterogeneity among the included studies, a bivariate mixed-effects model was used to evaluate the diagnostic performance of PAX1 methylation. As presented in Table 5, the pooled sensitivity and specificity were 0.87 (95% CI: 0.79–0.92) and 0.75 (95% CI: 0.52–0.89), respectively. The positive likelihood ratio (PLR) was 3.5 (95% CI: 1.6–7.4), the negative likelihood ratio (NLR) was 0.17 (95% CI: 0.10–0.29) and the diagnostic odds ratio (DOR) was 20 (95% CI: 7–62). A DOR greater than 1 suggests that PAX1 methylation is a strong discriminator for cervical cancer. Figures 6 and 7 illustrate the forest plots and the summary receiver operating characteristic (SROC) curve, with an area under the curve (AUC) of 0.89, indicating high overall diagnostic accuracy. The Q-test for sensitivity yielded a *p*-value of 0.04 and an I^2^ of 54.5%, indicating moderate heterogeneity. For specificity, the Q-test p-value was < 0.01, and the I^2^ was 97.65%, reflecting substantial heterogeneity among studies.

**Table 5.** Diagnostic indices of PAX1 methylation for cervical cancer screening.

Parameter	Estimate	95% CI
Pooled sensitivity	0.87	(0.79–0.92)
Pooled specificity	0.75	(0.52–0.89)
Pooled positive likelihood ratio	3.5	(1.6–7.4)
Pooled negative likelihood ratio	0.17	(0.10–0.29)
Pooled diagnostic odds ratio	20	(7–62)

#### 3.2.4. Influence assay and meta-regression

**Figure 8.**
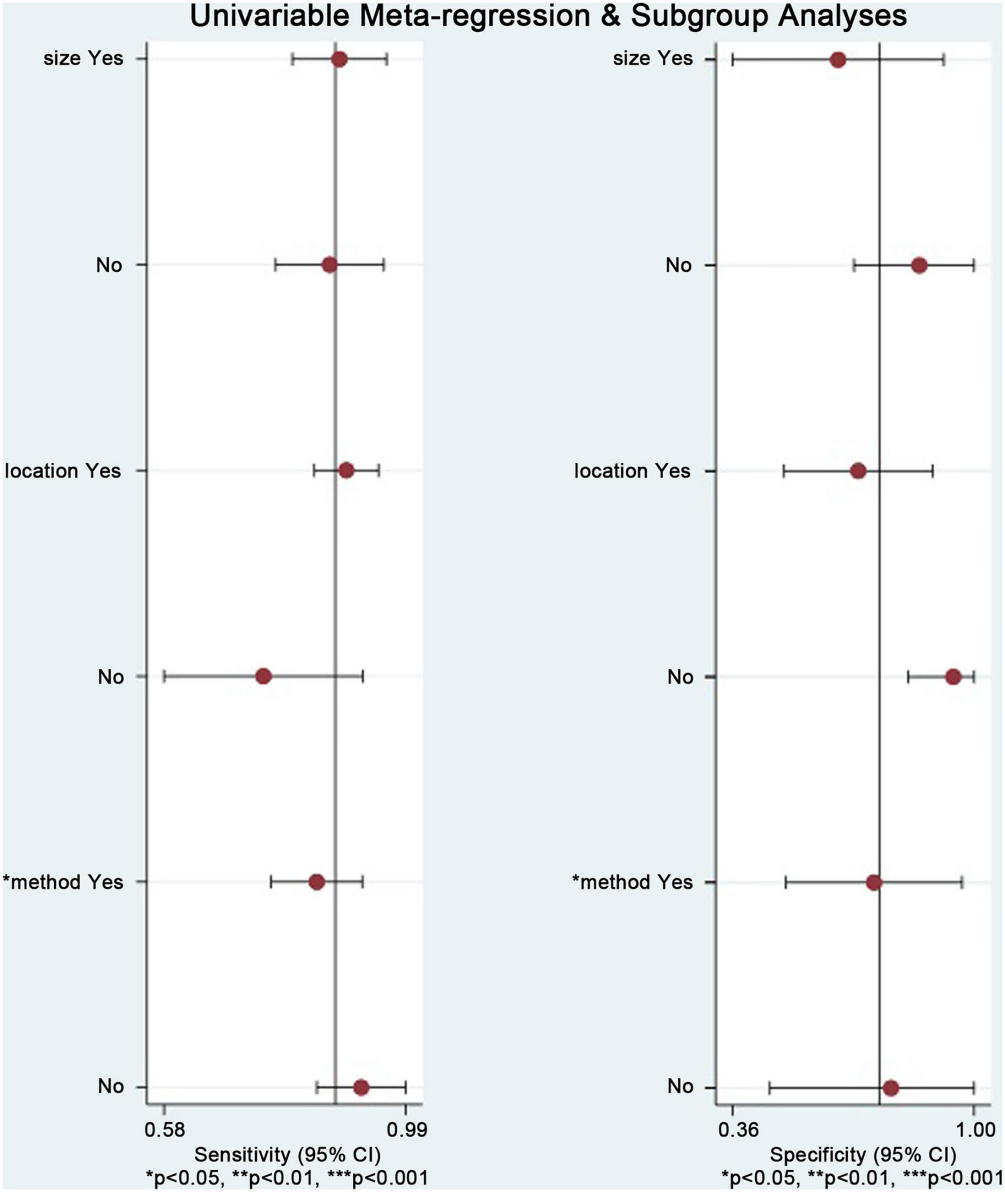
Influence and outlier detection analyses of the overall pooled study.

Due to the heterogeneity of non-threshold responses in this study, we performed an influence analysis and meta-regression to investigate the source of heterogeneity by assessing the effect of three predetermined covariates (sample size, study location, and test method) on sensitivity and specificity using Stata 16.0. As shown in Table 6 and Figure 8, only the test method variable in sensitivity was statistically significant (*p* < 0.05), indicating that the test method may be a source of heterogeneity.

**Table 6.** Meta-regression for the potential source of heterogeneity.

Stratified analysis	No. studies	Sensitivity (95% CI)	P_1_	Specificity (95% CI)	P_2_
Sample size					
≤200	4	0.88 (0.80–0.96)	0.18	0.64 (0.36–0.92)	0.19
>200	3	0.86 (0.77–0.95)		0.86 (0.68–1.00)	
Study location					
China	6	0.89 (0.84–0.95)	0.95	0.69 (0.50–0.89)	0.16
Korea	1	0.75 (0.58–0.92)		0.95 (0.82–1.00)	
Test method					
BSP	5	0.84 (0.76–0.92)	0.01	0.73 (0.50–0.97)	0.92
QMSP	2	0.92 (0.84–0.99)		0.78 (0.46–1.00)	

#### 3.2.5. Publication bias

**Figure 9.**
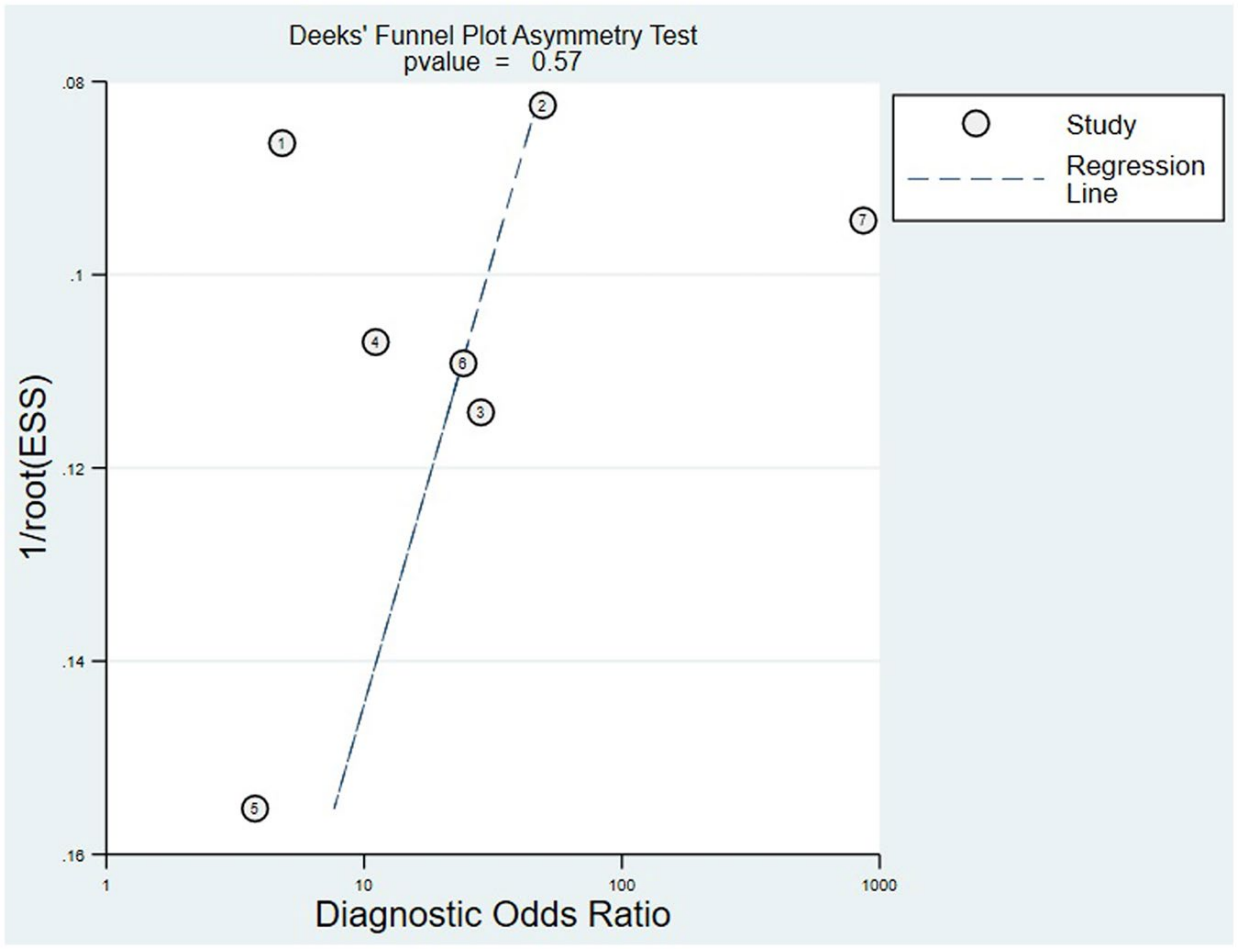
Funnel graph for the assessment of potential publication bias of the included studies. *p* = 0.57.

For the PAX1 methylation assay, the funnel plot for assessing publication bias showed no asymmetry with a *p*-value of 0.57, indicating no significant evidence of publication bias in the meta-analysis (Figure 9).

## 4. Discussion

DNA methylation is one of the most widely studied epigenetic modifications in mammals, regulating gene expression through silencing without altering the underlying DNA sequence. Aberrant methylation of CpG islands in the promoter regions of tumour suppressor genes is frequently observed in human cancers and is closely linked to tumour development [19]. The PAX gene family encodes transcription factors essential for cell differentiation and organ development. Among them, PAX1 functions as a tumour suppressor and is known to be epigenetically silenced in several malignancies, including ovarian cancer [20], oral squamous cell carcinoma [21] and oesophageal squamous cell carcinoma [22]. In particular, PAX1 methylation has shown promising sensitivity and specificity as a biomarker in cervical cancer.

Although HPV testing is highly sensitive in primary cervical cancer screening, its low specificity results in many false positives and unnecessary colposcopy referrals. TCT also suffers from limitations, with sensitivity for detecting HSIL and more severe lesions ranging between 50% and 70%, depending heavily on the reader’s expertise. In our study, the sensitivity of TCT for HSIL was only 16.95%. Previous studies have demonstrated that PAX1 gene methylation offers valuable diagnostic potential for cervical cancer [12,23,24]. In the current analysis, PAX1 methylation achieved AUCs of 0.83 (95% CI: 0.78–0.89) for HSIL and 0.92 (95% CI: 0.86–0.99) for CSCC. For HSIL, both sensitivity and specificity were 0.75, while for CSCC, the sensitivity was 0.81 and the specificity increased substantially to 0.94. This suggests that PAX1 methylation performs particularly well in identifying cervical cancer, although it is less effective in detecting HSIL.

To further assess the diagnostic performance of PAX1 methylation in cervical cancer, we conducted a comprehensive meta-analysis of studies published up to 1 September 2022. The pooled sensitivity and specificity were 0.87 and 0.75, respectively, with an AUC 0.89, indicating high overall diagnostic accuracy. The diagnostic odds ratio (DOR), which reflects the strength of association between test results and disease presence, was 20—well above the threshold of 1.0—confirming strong discriminatory power [25,26]. Additionally, the pooled PLR was 3.5, suggesting that patients with cervical cancer were 3.5 times more likely to test positive for PAX1 methylation compared to those without disease. The NLR was 0.17, indicating that a negative test result corresponded to a 17% false-negative rate, supporting its potential utility in ruling out high-risk cases.

Due to the observed heterogeneity not attributable to threshold effects, we explored potential sources using influence analyses and meta-regression. These analyses indicated that the detection method (e.g. BSP vs. QMSP) may be a source of inter-study heterogeneity. However, the analysis has limitations. Only seven studies were included, six of which were conducted in China. This geographic concentration introduces potential bias and limits generalizability. In our own clinical dataset, PAX1 methylation showed over 80% sensitivity and specificity for detecting CSCC, possibly reflecting regional diagnostic performance in the south-western part of Shandong Province, China. To determine the broader applicability of PAX1 methylation as a universal screening biomarker, future studies are needed in more diverse populations, including those from Europe, the Americas, and Africa.

This study confirms the high diagnostic performance of PAX1 methylation in cervical cancer screening. However, its potential role in guiding clinical management of women with HSIL, particularly in conservative approaches such as active surveillance (‘wait-and-see’), requires further investigation. Preliminary evidence suggests [27] that dynamic changes in PAX1 methylation levels may reflect lesion behaviour, with decreasing values after diagnosis associated with spontaneous regression, while persistently high levels may indicate on-going disease activity and the need for treatment. Nevertheless, high-quality prospective studies are lacking to confirm the utility of PAX1 methylation for monitoring disease progression or regression over time. To support the personalized management from screening to treatment, future research should aim to develop predictive models that integrate multi-omics data (e.g. transcriptomics, proteomics).

## 5. Conclusion

This study supports the clinical utility of PAX1 methylation as a promising biomarker for cervical cancer screening. The findings indicate that PAX1 methylation may enhance diagnostic accuracy, reduce unnecessary colposcopic procedures, and offer a noninvasive screening alternative. However, given the limited sample size and geographic concentration of included studies, further large-scale and multicentre validation, particularly in diverse populations, is needed to confirm its diagnostic performance and potential role in personalized clinical management.

## Data Availability

The data that support the findings of this study are available from the corresponding author upon reasonable request. The data are not publicly available due to privacy or ethical restrictions.
